# Expression of bcl-2 and p53 in bovine cutaneous fibropapillomas

**DOI:** 10.1186/1750-9378-10-2

**Published:** 2015-01-12

**Authors:** Florentina Bocaneti, Gennaro Altamura, Annunziata Corteggio, Elena Velescu, Giuseppe Borzacchiello

**Affiliations:** Department of Public Health, Faculty of Veterinary Medicine, University of Agriculture Sciences and Veterinary Medicine, Ion Ionescu de la, Brad, Iasi, Romania; Department of Veterinary Medicine and Animal Productions, University of Naples Federico II, Via F. Delpino, 1, 80137 Napoli, Italy

**Keywords:** Bovine papillomavirus, Cattle, Fibropapilloma

## Abstract

**Background:**

Bovine cutaneous fibropapillomas are benign hyperproliferative lesions induced by Bovine Papillomaviruses (BPVs). Bcl-2 is an important anti-apoptotic protein which is expressed in several cancer types. In contrary, p53 is a tumour suppressor protein that mediates cell cycle arrest, apoptosis and senescence in response to cellular stresses.

**Findings:**

Here, we investigated immunohistochemically and biochemically, the expression of bcl-2 and p53 in a subset of BPV positive fibropapillomas and bovine normal skin. Normal skin samples showed a weak signal for both proteins in the cytoplasm of the basal cells. Nine out of twelve (75%) tumour samples stained positive for bcl-2 throughout basal and parabasal layers, with most of cells showing strong cytoplasmic immunoreactivity. Nine out of twelve (75%) fibropapillomas were found to be positive for p53 expression, showing a strong cytoplasmic and perinuclear staining of p53 protein mainly in the basal and parabasal layers.

**Conclusions:**

Our data reveal an altered bcl-2 and p53 immunoreactivity in bovine cutaneous fibropapillomas, suggesting involvement of these two proteins in the cutaneous neoplastic transformation through an impaired apoptotic process.

**Electronic supplementary material:**

The online version of this article (doi:10.1186/1750-9378-10-2) contains supplementary material, which is available to authorized users.

## Background

Bovine cutaneous fibropapillomas are benign hyperproliferative lesions induced by different BPVs types, infecting both the epithelial cells and the underlying dermis [1]. These lesions usually undergo spontaneous regression, although in some cases the bovines might develop extensive papillomas resulting in debilitated animals that may succumb [1].

BPV infection is supposed to be the most important causal factor in fibropapilloma development and our previous studies on these tumours have demonstrated alterations of GAP junction proteins and signaling transduction pathways [2]. However, the mechanisms underlying cutaneous fibropapilloma development are not yet fully understood.

Papillomaviruses (PVs) are oncogenic viruses causing both benign and malignant tumours in humans as well as in several animal species. These viruses have developed numerous strategies in order to block host-mediated apoptosis, contributing to cancer development [3].

Programmed cell death, known as apoptosis, is a pivotal mechanism of cell death that plays an important role during diverse biological processes, including development, cell differentiation and proliferation [4]. The regulation of cells undergoing apoptosis is controlled by the bcl-2 proteins family; it includes both pro- (i.e. Bax, Bak) and anti-apoptotic proteins (i.e. bcl-2 itself) triggering or blocking, respectively, the release from mythocondria of proteins activating the apoptotic molecular cascade [4]. Generally, cells at proliferative stages express bcl-2, whilst it is downregulated in cells undergoing differentiation; indeed, bcl-2 is expressed in the germinal layer of epithelium, indicating a role in protecting the cells from apoptosis and providing a perpetual supply of cells for differentiation [5]. Several mechanisms have been found to cause bcl-2 dysregulation and cancer, including activating chromosomal translocation [6] and up-regulation upon viral infection: for instance, up-regulated bcl-2 expression has been described in different tumours, including premalignant and malignant lesions of the uterine cervix induced by Human Papillomaviruses (HPVs) [7–9]. In veterinary oncology, impaired bcl-2 expression has been recorded also in different feline epithelial tumours, such as basal cell tumour, carcinoma *in situ* and mammary carcinoma [10].

P53 is a tumour suppressor protein that mediates cell cycle arrest, DNA integrity and repairing, apoptosis and senescence in response to specific cellular stresses (e.g. various types of DNA damage, hypoxia, oncogene deregulation, oxidative damage) [11]. As a consequence of stress, p53 translocates from the nucleus to the cell cytoplasm where it directly interacts with and regulates members of the bcl-2 proteins family, including bcl-2 itself. This results in the release of various pro-apoptotic proteins, which in turn activate apoptosis [11].

P53 has a significant role in cell cycle control, therefore the loss of its suppressive function has been reported for different types of human neoplasia and animal tumours, such as BPV induced tumours [12–14]. However, studies concerning the expression and the impact of anti-apoptotic protein bcl-2 and the tumour suppressor p53 in BPV induced cutaneous tumours are lacking.

## Findings

**Figure 1:**
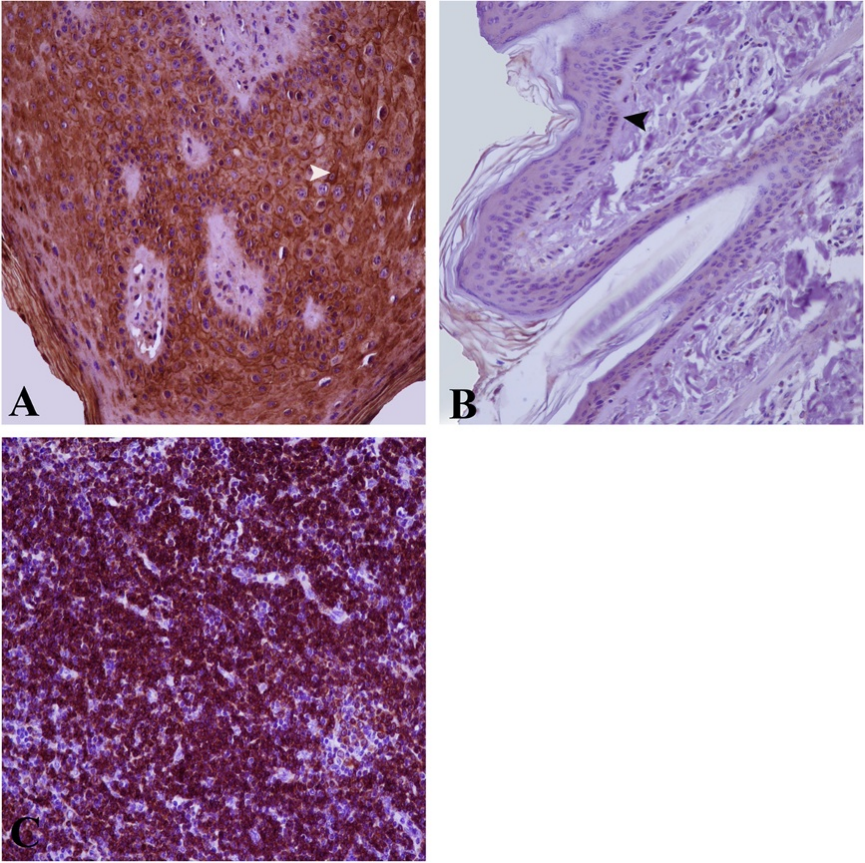
**Bcl-2 expression in bovine cutaneous fibropapillomas and normal skin; (A) bcl-2 is strongly expressed in the cytoplasm and membrane of the neoplastic cells of the basal and parabasal layers (white arrowhead); (B) Normal skin sample showing a weak expression of bcl-2 limited to the cytoplasm of the cells of basal layer (black arrowhead); (C) A positive control represented by human non- Hodgkin lymphoma showed a cytoplasmic immunoreactivity.** IHC X 200.

In this study, we investigated the expression of p53 and bcl-2 in bovine fibropapillomas according to the detailed “Materials and Methods” in the Additional file 1. Histopathologically, the tumours were all classified as cutaneous fibropapillomas. The PCR assay confirmed the presence of the BPV −1/-2 DNA in the tested samples, as detailed in Table 1. The expression of bcl-2 and p53 was assessed by immunohistochemistry and Western blot. Nine out of twelve (75%) fibropapillomas samples stained positive for bcl-2 throughout basal and parabasal layers, with most of cells showing strong cytoplasmic and membrane immunoreactivity (Figure 1A). The normal skin samples showed weak bcl-2 cytoplasmic staining, which was confined to the cells of basal layer (Figure 1B). The intensity and the expression pattern of each sample are summarized in Table 1. A human non- Hodgkin lymphoma used as positive control exhibited a strong cytoplasmic immunoreactivity (Figure 1C). Further, to check whether the expression of bcl-2 influences the rate of cell survival, we determined the apoptotic index (AI) comparing bcl-2 positive versus bcl-2 negative samples, as described in “Materials and Methods” section in Additional file 1. An AI of 43% (±5.2) was observed in bcl-2 negative fibropapillomas, whereas bcl-2 positive samples showed an AI of 37% (±2.57) (data not shown).

**Figure 2:**
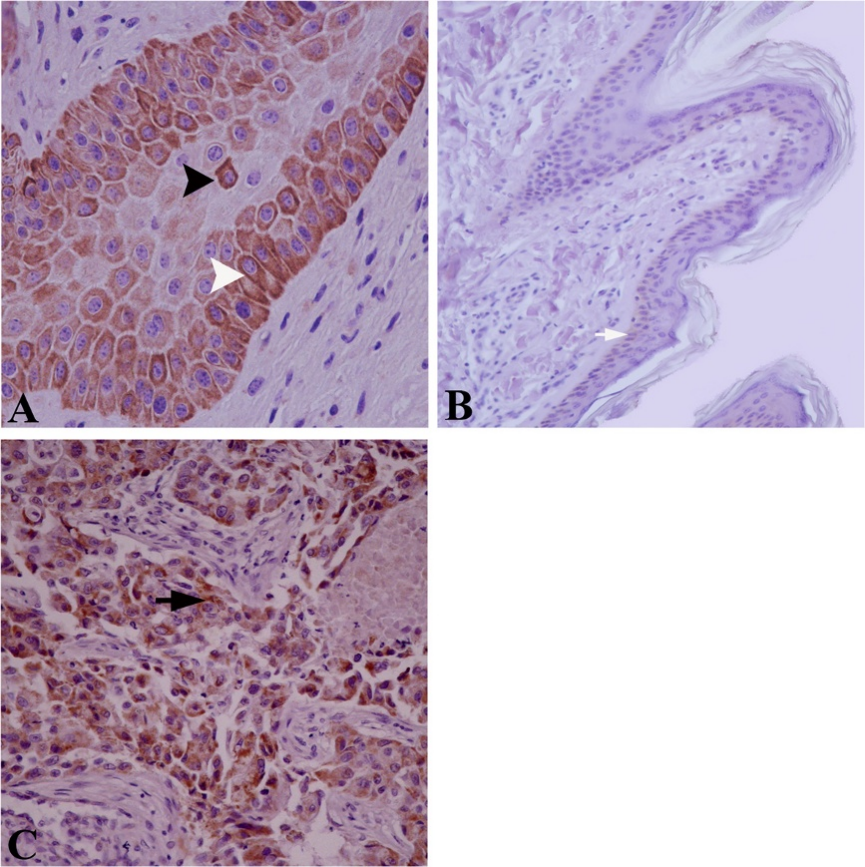
**P53 expression in bovine cutaneous fibropapillomas and normal skin; (A) p53 is expressed in the cytoplasm of basal (white arrowhead) and parabasal epithelial cells; note a perinuclear pattern in some cells of the parabasal layer (black arrowhead); (B) Normal skin samples showing a weak p53 cytoplasmic staining, confined to the basal cell layer (white arrow); (C) Epithelial cells of human mammary cancer showed cytoplasmic immunoreactivity (black arrow).** IHC, A X 400, B, C X 200.

**Figure 3:**
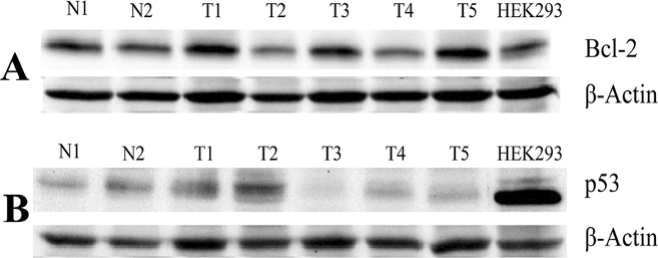
**Western blotting for bcl-2 and p53 proteins in bovine cutaneous fibropapillomas and normal skin.** **(A)** Western blot analysis of bcl-2 in bovine normal skin and fibropapillomas (upper blot); the blot was stripped and reprobed with anti-β-actin antibody to confirm equal loading of proteins in each lane (lower blot). **(B)** Western blot analysis of p53 in bovine normal skin and fibropapillomas (upper blot); the blot was stripped and reprobed with anti-β-actin antibody to confirm equal loading of proteins in each lane (lower blot).

Nine out of the 12 (75%) bovine fibropapillomas were found to be positive for p53 expression. In most samples, a strong expression of p53 protein was detected in the basal layer and parabasal layer, where cells with a cytoplasmic and perinuclear staining were recorded (Figure 2A). In the spinous and granular layers, few cells with an intense perinuclear staining were observed. In normal skin samples, the expression of p53 was weak and limited to the cytoplasm of the cells of basal layer (Figure 2B). The positive control represented by human mammary cancer showed a cytoplasmic staining (Figure 2C). To check the specificity of the antibodies used throughout the study, Western blot analysis was performed, showing a band of the expected size for bcl-2 (25KDa) and p53 (53KDa) in the tested samples as well as in HEK293 cell line used as positive control (Figure 3A, 3B).

**Table 1:** **BPV 1/2 DNA presence and bcl-2 and p53 protein expression in normal skin and bovine cutaneous fibropapillomas**

Sample	BPV 1/2 DNA	Bcl-2			p53		
		Staining	Pattern	Intensity	Staining	Pattern	Intensity
T1	+	Yes	Cytoplasmic, Membranous	+++	Yes	Cytoplasmic Perinuclear	++
T2	+	Yes	Cytoplasmic, Membranous	+++	Yes	Cytoplasmic Perinuclear	+++
T3	+	Yes	Cytoplasmic, Membranous	++	Yes	Cytoplasmic Perinuclear	++
T4	+	Yes	Cytoplasmic, Membranous	++	Yes	Cytoplasmic Perinuclear	+++
T5	+	Yes	Cytoplasmic, Membranous	+++	Yes	Cytoplasmic Perinuclear	+
T6	+	No	No	-	Yes	Cytoplasmic Perinuclear	+
T7	+	Yes	Cytoplasmic	+++	Yes	Cytoplasmic	+
T8	+	Yes	Cytoplasmic, Membranous	++	No	No No	-
T9	+	No	No	-	No	No No	-
T10	+	Yes	Cytoplasmic, Membranous	+	Yes	Cytoplasmic Perinuclear	++
T11	+	No	No	-	No	No	-
T12	+	Yes	Cytoplasmic	++	Yes	Cytoplasmic Perinuclear	+++
N1	+	Yes	Cytoplasmic	+	Yes	Cytoplasmic	+
N2	+	Yes	Cytoplasmic	+	Yes	Cytoplasmic Perinuclear	++

The presence of BPV 1/2 DNA is indicated by “+”. The cellular pattern of the immunoreactivity is either indicated with cytoplasmic and/or membranous and/or perinuclear. The intensity of immunolabelling for each specimen was scored in a “blind study” on a four-tiered scale: −, absent; +, weak signal or very weak signal; ++, moderate signal; +++, strong signal. “T” stands for tumour samples and “N” stands for normal skin samples.

By immunohistochemistry, two fibropapilloma samples (T9 and T11) did not expressed neither bcl-2 nor p53 proteins. The lack of reactivity might be due to the loss of antigenicity.

Bovine cutaneous fibropapillomas are benign lesions known to affect cattle worldwide. BPV are considered as causative agents of these tumours, inducing the proliferation of both epithelial and dermal cells [1]. Apoptosis is a tightly regulated process that plays an important role in development and homeostasis, responsible for the balance between cell proliferation and cell death, where bcl-2 has been proven to have a central role [6].The deregulation of the normal apoptotic process is considered as an “hallmark” of cancer [5, 7, 15, 16]. In our study we showed that in normal skin bcl-2 protein expression is confined to the cytoplasm of the basal cell layer, which is in agreement to that described in feline, canine and human skin [10, 17]. The basal keratinocyte-restricted expression of bcl-2 suggests that it may protect the cells from apoptosis, being responsible for the proliferation and regeneration of the epithelium [5]. Bcl-2 expression in the cytoplasm and plasma membrane specifically inhibits the cell death, resulting in cell surviving [18]. In BPV positive fibropapillomas, a strong immunoreactivity of bcl-2 was observed in all layers. This is the first time, to the best of our knowledge that bcl-2 is reported to be expressed in the membrane and the cytoplasm of bovine neoplastic cells, suggesting an active pathogenic contribution in protecting these cells by inhibiting the normal apoptotic processes. Similarly, a relatively high bcl-2 expression was recorded in HPV associated epidermodysplasia verruciformis, where the interaction between oncogenic HPV and bcl-2 may result in increased susceptibility toward acquirement of somatic mutations and malignant transformation [16]. We speculate that bcl-2 deregulation maybe a common mechanism in PVs induced cancerogenesis.

In our study, bcl-2 positive samples showed a reduced number of TUNEL positive cells when compared to the bcl-2 negative fibropapillomas. This is in accordance with human counterpart, where in head and neck squamous cell carcinomas the number of apoptotic cells was noted to be reduced in bcl-2 positive tumours when compared to bcl-2 negative tumours [7]. This finding suggests that apoptosis might be regulated by bcl-2 expression also in BPV induced fibropapillomas. Bcl-2 upregulation is one of the potential mechanisms by which tumour cells escape from p53 mediated apoptosis; functional p53 inhibits bcl-2 expression and its anti-apoptotic function, thus altered localization and/or expression of p53 may lead to bcl-2 overexpression and exertion of its anti-apoptotic activity [8].

Normally, a very low expression of p53 protein is detected in cells; in response to cellular stresses, however, the protein is stabilized and cell division is inhibited in order to respond to such damage [19].

Our results indicate that p53 is weakly expressed in basal epithelial cells of normal skin samples. Accordingly, a similar p53 expression pattern in the basal epithelial cells has been recorded in human normal skin, suggesting that it may exert its physiological function in bovine healthy epithelium [15, 20, 21].

Fibropapilloma samples showed a strong cytoplasmic and perinuclear p53 expression in the basal and parabasal cell layers. Cytoplasmic accumulation of p53 has been proposed as a considerable mechanism to disrupt its function as a tumour suppressor and its expression has been reported in different human and animal tumours [22, 23]. Recent findings report cytoplasmic overexpression of p53 in BPV induced equine sarcoids; moreover, a perinuclear p53 expression has been reported in the same tumours, suggesting that p53 alteration contributes to disruption of the equilibrium of cell growth and cell death in BPV associated tumours in different species [12, 13, 24].

However, the higher expression of p53 in epithelial component of fibropapilloma may be due to different stresses followed by a temporary increase of the normal p53 protein or accumulation of mutated p53, resulting in a permanent abnormal form of p53 that is not functional and/or resistant to degradation [25].

Our results indicate that bcl-2 expression may promote the survival of the neoplastic cells possibly depending on p53 impaired expression.

Despite a number of studies have higlighted a role for BPV’s oncogenes in impairing some cellular pathways and leading to neoplastic transformation, nothing is known about a possible interplay among the viral oncoproteins and bcl-2 and p53 dysregulation. Thus, the unraveling of new pathways possibly involved in the pathogenesis of bovine cutaneous fibropapillomas is of a great importance to complete the molecular scenario triggering to the development of these tumours.

In summary, the current study provides evidence for p53 and bcl-2 expression in a majority of BPV-induced bovine tumours, suggesting an involvement of these two proteins in bovine cutaneous fibropapilloma development. Further studies are needed to better understand bcl-2 and p53 function *in vivo* carcinogenesis.

## Electronic supplementary material

Additional file 1: **Materials and Methods.** (DOCX 32 KB)
