# Supplementary material for: Expression of bcl-2 and p53 in bovine cutaneous fibropapillomas
Source: Infect Agent Cancer. 2015 Jan 12;10:2. doi: 10.1186/1750-9378-10-2 (PMC4298047; doi:10.1186/1750-9378-10-2)
Supplement: Supplementary file 1 — Additional file 1: Materials and Methods. (DOCX 32 KB) [file 13027_2014_512_MOESM1_ESM.docx]

**Materials and methods**

**Tumour samples**

A number of twelve fibropapilloma (T1-T12) and two normal skin (N1-N2) samples were collected for this work. Samples N1-N2 and T6-T12 were known to be BPV positive from a previous study reported by Silva et al. (2013). Samples T1-T5 were collected from cows suffering from naturally occurring cutaneous fibropapillomas from Moldova, Romania. Briefly, the tissue samples were divided in two parts: one part was stored at -80^0^ C for biochemical analysis and one part of each sample was fixed in 10% neutral buffered formalin and routinely embedded in paraffin. The samples were sectioned and stained with Haematoxylin & Eosin for histopathological assessment. The diagnosis was assessed following the guidelines proposed by Goldschmidt et al., 1998 and all the samples were diagnosed as cutaneous fibropapillomas.

**Polymerase chain reaction (PCR)**

Genomic DNA was extracted from the fibropapilloma samples T1-T5 using the DNeasy Blood and Tissue kit (Qiagen), in compliance with the manufacturer’s protocols.

PCR assay was performed using a Master Mix kit (Applied Biosystems) following the manufacturer’s instructions. Amplification of the E5 open reading frame (ORF) was carried using a BPV-1 and -2 consensus primer pair, BPV-E5 F (TTGCTGCAATGCAACTGCTG corresponding to BPV nucleotides 3915 to 3934) and BPV-E5 R (TCATAGGCACTGGCACGTT corresponding to BPV nucleotides 4208 to 4225) amplifying a fragment of 311 bp from nucleotide 3915 to 4225.

The reactions were performed in a total volume of 25μL, containing 50ng of DNA, 3 mM MgCl2, 200 μM of each dNTP, 0.25 U AmpliTaq Gold Polymerase (Applied Biosystems), and 0.5 μM of each oligonucleotide primer. Reaction conditions were: denaturation for 5 min at 94^0^ C, followed by 2 cycles of denaturation at 94^0^ C for 1 min, annealing at 55^0^ C and extension at 72^0^ C for 1 min and followed by 35 cycles of denaturation at 94^0^ C for 30 s, annealing at 52^0^ C for 30 s and extension at 94^0^ C for 30 s. Amplicons were separated by electrophoresis in 1% agarose gels with Tris acetate ethylene diamine tetraacetic acid (EDTA) buffer (TAE; 40 mM Tris, 1 mM Na_2_EDTA, 20 mM acetic acid), stained with ethidium bromide and visualized under ultraviolet light. A blank sample consisting of reaction mixture without DNA and a positive sample consisting of BPV-2 cloned genome (Roperto et al. 2012) were used.

**Immunohistochemistry**

Sections from the 12 fibropapilloma and the 2 normal skin samples were immunostained using a streptavidin-avidin method (LSAB Kit; Dako). Paraffin sections of 4μm thickness were deparaffinized in xylene and 100% ethanol. Endogenous peroxidase activity was blocked by 0.3% H_2_0_2_ methanol solution for 20 min. The sections were subjected to antigen retrieval with sodium citrate (pH 6.00) by heating in a microwave twice for 5 min each cycle (at 700W) and allowed to cool for 10 min. Sections were rinsed twice for 5 min in phosphate buffer saline (PBS; pH 7.4, 0.1 M). To block non-specific bindings protein block serum (Dako) was applied for 15 min. The mouse anti bcl-2 (NCL-bcl-2-486, Novocastra) primary antibody diluted 1:50 in PBS was applied overnight at 4^0^ C in humified chamber, while the mouse anti-p53 primary antibody (NCL-p53-505, Novocastra) diluted 1:50 in PBS was applied for 1h at room temperature (RT) in humified chamber. Further, the sections were incubated for 20 min at RT with the appropriate biotinylated secondary antibody. Following a rinsing step in PBS, streptavidin-conjugated to horseradish peroxidase was applied for 20 min at RT. Colour development was obtained by treatment with diaminobenzidine (DAB) (Dako) for 5 min. Sections were counterstained with Mayer’s haematoxylin. In the corresponding negative control section, the primary antibody was either omitted or replaced with appropriate normal serum. The scoring of the immunoreactivity was determined in a “blind” study by two observers (GB and FB). The intensity of labelling in each specimen was scored from absent to very strong immunosignal. Positive controls consisted of paraffin embedded sections of human non- Hodgkin lymphoma for bcl-2 and human mammary cancer for p53 (kindly provided by dr. Carlo della Ragione, AORN - A. Cardarelli – Napoli).

**Western blot analysis**

Molecular analysis was performed on two normal skin samples (N1–N2) and on five tumour samples (T1–T5). The samples were lysed in ice-cold RIPA buffer (50mM Tris-HCl, pH 7.5, 150 mM NaCl, 1%Triton X100, 1mM EDTA, deoxycholate 0,25%) added with phosphatase inhibitor cocktails and protease inhibitor cocktail (Sigma, Italy). Protein concentrations were determined by use of a protein assay kit (Bio-Rad Laboratories). Equal amounts of lysate were resuspended in Laemmli sample buffer (sodium dodecyl sulphate (SDS), Tris-HCl pH 6.8, glycerol, bromophenol blue, and 2*β*-mercaptoethanol), boiled and analyzed by SDS polyacrylamide gel electrophoresis (PAGE) and immunoblotting. Nitrocellulose membranes were blocked with 5% bovine serum albumin (BSA)-in-Tris-buffered saline (TBS: 12.5

mM TrisHCl pH 7.4; 125

mM NaCl) at RT and incubated over night at 4°C with mouse anti-bcl-2 (NCL-bcl-2-486, Novocastra) and mouse anti-p53 (NCL-p53-505, Novocastra) antibodies diluted 1

:

1000 and 1

:

500, respectively. After washing steps in TBS-Tween 0.1%, anti-mouse peroxidase-conjugated secondary antibody (Amersham, Gel Health Care) was applied 1 h at RT at 1

:

2000 dilution. Membranes were washed again, and bound antibodies were visualized by enhanced chemiluminescence (ECL) (LiteAblot Plus, EuroClone) using ChemiDoc gel scanner (Bio-Rad Laboratories) equipped with a densitometric workstation (Image Lab software, Bio Rad Laboratories). The membranes were stripped and reprobed against mouse anti-β-actin antibody (CP01, Calbiochem) at a dilution of 1:5000 to confirm equal loading of proteins in each lane. Whole cell lysate from HEK 293 cell line was run in parallel as positive control of p53 and bcl-2 immunoreactivity (Esposito et al. 2012).

**In situ detection of fragmented DNA (TUNEL assay)**

DNA degradation in the bovine cutaneous fibropapillomas was detected using the TACS 2 TdT-DAB (TREVIGEN) *in situ* apoptosis detection kit according to the manufacturer’s instructions. The fibropapilloma samples negative for bcl-2 expression and samples bcl-2 positive were stained. The paraffin sections were incubated for 5 min at 57^0^ C, deparaffined in xylene and hydrated in a graded series of ethanol before incubation with PBS for 10 min. Then, to make the DNA accessible to the enzymes, the cell membranes were permeabilized with Proteinase K for 20 min. The endogenous peroxidase activity was quenched using 30% H_2_O_2_ in methanol and washed 2 times in PBS. Next, the slides were immersed in TdT labeling buffer for 5 min. The labeling reaction mix was added into each section and incubated at 37^0^ C for 1 h in a humidity chamber. To stop the labeling reaction, the slides were immersed in TdT Stop Buffer for 5 min and rinsed in deionized water for 2 times. The samples were incubated with Strep-HPR solution for 10 min at 37^0^ C and rinsed two times in PBS. Visualization of peroxidase was carried out by immersing the slides in DAB solution for 5 min, followed by two washes in deionized water. The sections were counterstained with Mayer’s haematoxylin, then dehydrated in a series of ethanol, cleared in xylene and mounted for evaluation by light microscopy. The specificity of TUNEL technique was verified by using a negative control represented by a fibropapilloma section treated with the labeling reaction mix without the TdT Enzyme, while the positive control was represented by the same sample, treated with TACS-Nuclease to generate DNA breaks in every cell.

The percentage of apoptotic cells in tissue sections was defined as apoptotic index (AI) and analyzed by staining for DNA fragmentation, a characteristic marker of apoptosis. Slides were read and scored for the number of terminal deoxynucheotidyl transferase-mediated nick end labeling positive cells per 10 fields at 400 magnification for each sample by two independent observers.

Apoptotic index was calculated according to Kikuchi and NishiKawa (1997) as follows:

Apoptotic index (%) = Number of TUNEL positive cells X 100/ Total number of cells counted.

References

[Esposito, F](http://www.ncbi.nlm.nih.gov/pubmed?term=Esposito%20F%5BAuthor%5D&cauthor=true&cauthor_uid=22932725)., [Tornincasa, M](http://www.ncbi.nlm.nih.gov/pubmed?term=Tornincasa%20M%5BAuthor%5D&cauthor=true&cauthor_uid=22932725)., [Federico, A](http://www.ncbi.nlm.nih.gov/pubmed?term=Federico%20A%5BAuthor%5D&cauthor=true&cauthor_uid=22932725)., [Chiappetta, G](http://www.ncbi.nlm.nih.gov/pubmed?term=Chiappetta%20G%5BAuthor%5D&cauthor=true&cauthor_uid=22932725)., [Pierantoni, G.M](http://www.ncbi.nlm.nih.gov/pubmed?term=Pierantoni%20GM%5BAuthor%5D&cauthor=true&cauthor_uid=22932725)., [Fusco, A](http://www.ncbi.nlm.nih.gov/pubmed?term=Fusco%20A%5BAuthor%5D&cauthor=true&cauthor_uid=22932725)., 2012. High-mobility group A1 protein inhibits p53-mediated intrinsic apoptosis by interacting with Bcl-2 at mitochondria. Cell Death and Disease. 3:e383. doi: 10.1038/cddis.2012.126.

Goldschmidt, M.H., Dunstan, R.W., Stannard, A.A., von Tscharner, C., Walder, E.J., Yager, J.A., 1998. Histological Classification of Epithelial and Melanocytic Tumors of the Skin of Domestic Animals. World Health Organization International Histological Classification of Tumors of Domestic Animals, Second Series, vol. III. Armed Forces Institute of Pathology, American Registry of Pathology, Washington, DC, USA, 106pp.

[Kikuchi, A](http://www.ncbi.nlm.nih.gov/pubmed?term=Kikuchi%20A%5BAuthor%5D&cauthor=true&cauthor_uid=9236520)., [Nishikawa, T](http://www.ncbi.nlm.nih.gov/pubmed?term=Nishikawa%20T%5BAuthor%5D&cauthor=true&cauthor_uid=9236520)., 1997. Apoptotic and proliferating cells in cutaneous lymphoproliferative diseases. Archives of Dermatology 133(7), 829-33.

Roperto, S., Borzacchiello, G., Esposito, I., Riccardi, M., Urraro, C., Luca, R., Corteggio, A., Tate, R., Cermola, M., Paciello, O., Roperto, F., 2012. Productive infection of bovine papillomavirus type 2 in the placenta of pregnant cows affected with urinary bladder tumors. PLoS One 7, e33569.

Silva, M.A, Altamura,G., Corteggio, A., Roperto, F., Bocaneti, F., Velescu, E., Freitas, A.C., Carvalho, C.R., Cavalcanti, , P.S, Borzacchiello, G., 2013. Expression of connexin 26 and bovine papillomavirus E5 in cutaneous fibropapillomas of cattle. Veterinary Journal 195, 337-343.
